# Trajectories of Symptom Clusters and Their Predictive Factors in Patients With Colorectal Cancer 3 Months After Surgery: A Longitudinal Study

**DOI:** 10.1002/cam4.71025

**Published:** 2025-07-14

**Authors:** Yue Li, Wenwen Gan, Qin Mao, Hongying Wu, Ting Cao, Haiyan Wu, Xiaorong Mao

**Affiliations:** ^1^ Department of Nursing, Sichuan Provincial People's Hospital, School of Medicine University of Electronic Science and Technology of China Chengdu China

**Keywords:** colorectal neoplasms, convalescence, longitudinal studies, perioperative period, predictive factors, subgroup analysis, symptom clusters, trajectories

## Abstract

**Aims:**

To examine the changing trajectories of symptom clusters within 3 months following surgery in patients with colorectal cancer (CRC) and identify their predictive factors.

**Design:**

A prospective longitudinal observational study.

**Methods:**

Convenience sampling was used to recruit inpatients with CRC who were scheduled for surgical treatment at the Sichuan Provincial People's Hospital between October 2022 and September 2023. The Chinese version of the MD Anderson Symptom Inventory Gastrointestinal Cancer Module was utilized. The prevalence and severity of patients' symptoms were assessed at 7 days (T1), 6 weeks (T2), and 3 months (T3). Before the operation, a total of 240 patients with CRC were recruited. There were 203, 164, and 139 patients participating in T1, T2, and T3, respectively. Exploratory factor analysis identified symptom clusters. Latent class growth modeling determined the developmental trajectories of symptom clusters, and binomial logistic regression analyzed predictors of trajectory classification.

**Results:**

Two clinical subgroups were identified: a “high symptom—decreases and then increases” (17.2%) and a “low symptoms—continuous decline” (82.8%). The latter exhibited significantly lower and progressively declining symptom severity compared to the former. Predictive factors for the “high symptom—decreases and then increases” subgroup included multimorbidity (≥ 2 chronic conditions), chronic lung disease, preoperative frailty, severe anxiety/depression, open surgery, and postoperative chemotherapy.

**Conclusions:**

Targeted management of the mood‐sleep disorder cluster (T1) and the activity intolerance cluster (T2/T3) may significantly improve patients' quality of life. The “high symptom—decreases then increases” subgroup warrants prioritized clinical attention, as early intervention for its predictive factors (e.g., frailty, anxiety, and depression) could enhance postoperative outcomes. Integrating these factors into routine preoperative assessments is recommended.

## 1 Introduction

Colorectal cancer (CRC) ranks among the most prevalent malignancies globally, exhibiting both high incidence and mortality rates that contribute to its growing disease burden [1]. Surgery is the main treatment for CRC, and it varies according to tumor location, pathological stage, and operation technique. From 1 week to 3 months postoperatively, many patients require adjuvant chemoradiotherapy, such as hyperthermic intraperitoneal chemotherapy (HIPEC) combined with radiochemotherapy. It can be seen that the recovery period of CRC patients undergoing surgery has certain variability and complexity. Extensive research has consistently identified the 3‐month postoperative phase as the period of most pronounced symptom burden [2, 3]. Implementing targeted management strategies focused on early symptom intervention not only enhances patients' quality of life but may also yield positive impacts on long‐term prognosis [4].

The concept of Enhanced Recovery After Surgery (ERAS) has gained widespread adoption in recent years. This multimodal approach focuses on optimizing patients' psychological status, physical frailty, and activity levels during the perioperative period to significantly improve early postoperative recovery. In a meta‐analysis of 38 studies, ERAS pathways were found to be effective in reducing hospital length of stay (LOS) and postoperative complication rates [5]. The implementation of ERAS protocols, combined with advances in surgical techniques, has effectively reduced hospital stays, with some patients achieving discharge within 7 days post‐operation. However, during the post‐discharge self‐care phase, patients often face heightened uncertainty. This psychological burden not only exacerbates anxiety but may also trigger a series of physiological issues, including limitations in daily activities. Experts recommend that patients be followed up every 3 months within 1 year after surgery [6]. Moreover, delayed assessment may result in unresolved symptoms within the initial 3 months post‐surgery. During this critical transition period, patients must cope with both the physical and emotional impact of major surgery while adapting to multiple environmental shifts—from hospital to home and community settings. Studies indicate that many patients struggle to access timely medical support for postoperative symptoms, often resorting to passive waiting or self‐management based on personal experience [7, 8]. Therefore, conducting an in‐depth investigation into the symptom evolution and influencing factors among patients with CRC within 3 months post‐surgery helps alleviate patient suffering and provides a scientific basis for improving the hospital‐community collaborative nursing service management system, ensuring seamless transitions across medical settings.

Symptoms are subjective experiences that reflect changes in an individual's biopsychosocial functioning, sensation, or cognition [9]. Their assessment mainly relies on patient self‐reporting and has emerged as a critical concern for medical professionals. Importantly, symptoms rarely occur in isolation; instead, they often coexist as interrelated clusters with synergistic effects, resulting in an exponentially greater patient impact. Multiple symptoms may share common influencing factors or generating mechanisms [10]. Repeated studies focusing on individual symptoms waste medical resources and increase patient distress and burden. In 2001, Dodd introduced the concept of symptom clusters in oncology [11]. Scholars later defined symptom clusters as stable groupings of two or more symptoms that appear simultaneously and are interrelated [11]. Different symptom clusters are independent, and most symptom clusters change dynamically over time [12, 13]. Symptom cluster research promotes the exploration of common pathogenesis among symptoms by grasping the holistic view of humans and helps medical personnel implement management measures accurately and efficiently through simplified models, thereby conserving medical resources and reducing patient burden. With further research, symptom management has shifted from single symptoms to symptom clusters.

The Dynamic Model of Symptom Management [14] highlights the linear relationship between time and chronic diseases. Specifically, when a disease is in a state of recovery, deterioration, or stability, its changes can be abstracted as a dynamic trajectory that exhibits an upward trend, downward trend, or maintenance at a certain level. Simultaneously, the dimensions of symptoms also evolve over time. Interventions and adjustments to influencing factors—such as individual attributes, environmental conditions, and health characteristics—can positively alter these trajectories and enhance patient outcomes. Targeted interventions modifying influential factors—including individual attributes, environmental conditions, and health characteristics—can actively reshape these trajectories to improve patient outcomes. However, current studies on CRC symptom clusters were mostly cross‐sectional [15, 16, 17], failing to address the long‐term and complex nature of tumor treatment. These methods cannot accurately identify symptom clusters affecting patients' quality of life or reveal their temporal trends. Therefore, it is not conducive for health workers to implement prospective symptom management for patients with CRC with different characteristics in a targeted and phased manner. Longitudinal studies can compensate for this deficiency. While some researchers have examined symptom clusters in patients with CRC preoperatively or during the chemotherapy period [2, 18, 19, 20, 21], they have ignored symptom changes in specific post‐surgical periods, which may require more medical support, including medium‐ and long‐term care, home care services, etc. In previous studies, symptom clusters in patients with other diseases present different changes at different stages, and the clinical subgroups divided according to their change trajectory can provide a basis for the development of prospective interventions [22]. Therefore, latent class growth modeling (LCGM) was used in this study to explore the classification and predictive factors of symptom cluster trajectories in patients with CRC 3 months postoperatively, aiming to provide references for clinical management.

## 2 Methods

### 2.1 Design and Setting

This was a prospective longitudinal observational study. Patients with CRC who underwent surgery in the inpatient department of Sichuan Provincial People's Hospital from October 2022 to September 2023 were recruited using a convenience sampling method and surveyed 7 days, 6 weeks, and 3 months postoperatively.

### 2.2 Sample

**FIGURE 1:**
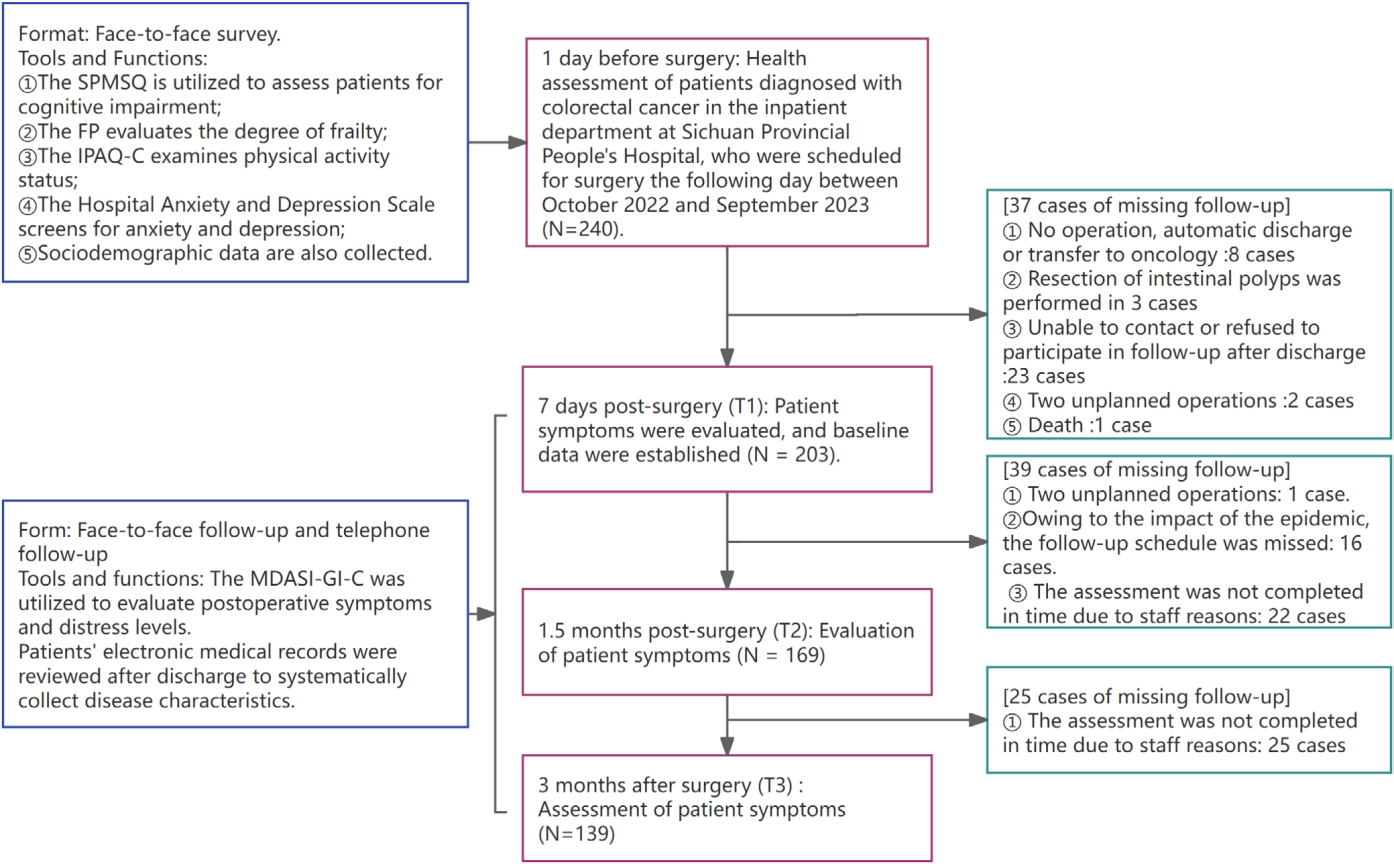
Flowchart and analysis of lost visits.

In this study, 203 people provided baseline data at T1, 164 people completed the follow‐up at T2, and 139 people completed all follow‐up components at T3 (Figure 1). Patients who met the following criteria were included in this study: (1) diagnosed with CRC and undergoing open or laparoscopic surgery; (2) age ≥ 18 years; (3) conscious and able to communicate with others without difficulty; and (4) volunteered to participate in this study and signed an informed consent form. Patients who met the following criteria were excluded: (1) severe cognitive dysfunction as screened by the Short Portable Mental Status Questionnaire (SPMSQ); (2) participation in other clinical studies; and (3) ileostomy closure performed before T3 follow‐up.

Exploratory factor analysis (EFA), an LCGM, and logistic regression analysis were used in this study. The minimum sample size for EFA is typically recommended to be 5–10 times the number of items, with a minimum requirement of at least 100 participants [23]. The LCGM follows the same sample size requirements as structural equation modeling (SEM), and it is generally considered that 100–150 participants are the minimum threshold for reliable parameter estimation [24]. In logistic regression analysis, the sample size should generally be at least 10 times the size of the smaller class of the dependent variable for robust model performance [25]. Therefore, the sample size of this study was set at least 100.

### 2.3 Assessment Tools

In this study, relevant variables were initially screened out through a literature review [3, 15, 20]. Subsequently, experts in this field were invited to modify the variables, and finally, a questionnaire including demographic characteristics and clinical characteristics was formed. Demographic characteristics included age, sex, and marital status. Clinical characteristics included cancer stage, pathological diagnosis, and type of surgery.

The Chinese version of the MD Anderson Symptom Assessment Scale Gastrointestinal Module (MDASI‐GI‐C) was used to assess symptom severity in patients with CRC. Developed in 2010 by MD Anderson Cancer Center, this validated instrument assesses symptoms and distress in patients with gastrointestinal cancer within 24 h [26]. The questionnaire consisted of three components: Part 1 contained 13 core symptom entries, Part 2 contained 5 gastrointestinal tumor‐specific symptom entries, and Part 3 contained 6 symptom distress entries. All items are rated on a 0–10 Likert scale, with higher scores indicating greater symptom severity or distress. The MDASI‐GI‐C demonstrates strong reliability and validity, with a Cronbach's *α* coefficient of over 0.8 for overall symptom severity and interference with daily living items [27].

The Hospital Anxiety and Depression Scale (HADS), developed by Zigmond and Snaith in 1983, was administered to evaluate patients' psychological distress. The scale consists of 14 items subdivided into 2 subscales—7 items each for anxiety and depression—and employs a 4‐point Likert scoring system. A total score of ≥ 11 suggests the presence of potentially significant symptoms of anxiety and depression [28]. The HADS is suitable for assessing mental well‐being in hospital settings, community environments, and primary care practice [29]. The overall Cronbach's *α* coefficient for general hospital inpatients in China was reported to be 0.785, indicating satisfactory internal consistency of the scale [30].

The frailty phenotype (FP) was used to assess frailty. The FP was developed by Fried et al. in 2001 and is widely used in assessing frailty in hospitalized patients [31]. The scale included five dimensions: body mass loss, 4.5‐m walking time, grip strength, physical activity, and self‐reported fatigue. Each dimension has a “yes” or “no” result: a “yes” was 1 point, and a “no” was 0 points. When the sum of the scores of the above five items is 0 points, there is no frailty; points 1–2 indicate pre‐frailty, and ≥ 3 indicate frailty.

The Chinese version of the International Physical Activity Questionnaire (IPAQ‐C) was used to evaluate patients' physical activity levels. Developed in 2001 by the International Physical Activity Measurement Working Group, this scale comprises seven questions that assess the frequency of heavy physical activity, moderate physical activity, and walking, as well as the duration of each intensity level, including sedentary behavior, to determine the extent of patients' physical activity [32]. Macfarlane et al. verified the reliability and validity of the IPAQ‐C in their study [33].

The SPMSQ, developed by Pfeiffer et al. in 1975, was utilized to screen and exclude patients with severe cognitive impairment [34]. This questionnaire includes 10 questions to evaluate cognitive function. Patients with less than elementary school education, those with middle school education, and those with high school education or above are considered to have severe cognitive impairment if they can correctly answer at most one question, two questions, and three questions, respectively. The retest reliability of the SPMSQ ranged from 0.8 to 0.83, demonstrating its satisfactory stability [35].

### 2.4 Data Collection

After potential participants were identified by the physician, informed consent was obtained from the patient by a registered nurse, following ample time for the patient to read the consent form and ask questions. Then, cognitive function, frailty, physical activity, anxiety, and depression of patients were assessed, and basic patients' information was collected. Patients' symptoms were dynamically assessed at three time points post‐surgery: 7 days (T1), 6 weeks (T2), and 3 months (T3), using a combination of telephone follow‐ups and face‐to‐face interviews. Data pertaining to patients' diseases were extracted from electronic medical records. Additionally, detailed data related to patients' conditions were extracted from the hospital's electronic medical record system. A pilot study was conducted before the main survey, and the questionnaire was sorted out, analyzed, and corrected in a timely manner.

It is considered that some CRC patients may have surgery canceled because of specific test results after admission. In this study, we collected data the day before surgery rather than the day of admission, which can minimize sample loss due to changes in treatment plans and may reduce the impact of treatment uncertainty on patient anxiety and depression. Moreover, considering the inherent variability of the disease, some patients may initiate intraperitoneal hyperthermic chemotherapy or other adjuvant therapies within 7 days post‐surgery. Concurrently, advancements in surgical techniques and the implementation of ERAS protocols have significantly reduced the duration of postoperative hospital stays, allowing some patients to be discharged within 7 days after surgery. Consequently, the heterogeneity in the subjective experiences of CRC patients becomes particularly pronounced during this 7‐day period post‐surgery, making it an optimal timeframe for capturing individual differences in patient baseline data. A literature review shows discrepancies in postoperative follow‐up timing for CRC across studies (e.g., 4, 6 weeks, after surgery, etc.) [2, 19, 36]. Most patients start chemotherapy around 4 weeks post‐surgery, but some delay it due to their physical condition. Some undergo stoma reversal surgery around 3 months. To assess the impact of chemotherapy while minimizing confounding factors and sample loss, the study was conducted 6 weeks post‐surgery. At this point, patients were discharged, so a hybrid approach of phone follow‐ups and face‐to‐face visits was used.

The study implemented rigorous quality control throughout the investigation. All investigators received standardized training on research protocols, assessment methods, and equipment use, with competency certification required prior to participation. During the investigation process, to ensure that patients accurately understood the requirements, the investigator was present while participants completed the questionnaire. When measuring grip strength and walking speed, the instruments were calibrated and set to zero prior to use to minimize measurement bias. When collecting questionnaires, verify the completeness of the filling, code them in a timely manner, and supplement missing items or correct incorrect data. Two people independently enter and verify the data to ensure its accuracy. A double‐blind design was employed: symptom assessment and medical record collection were conducted by an independent team, and the data from both parties were not shared. After anonymization, all the data were processed by the statistical analysis team using only desensitized data.

### 2.5 Data Analysis

#### 2.5.1 Statistical Description and Preprocessing

IBM SPSS version 26.0 and Mplus 8.0 were used for statistical analyses. Continuous variables were presented as means and standard deviations, and categorical variables were expressed as frequencies and percentages. The missing values for continuous variables are imputed using the mean, while the missing values for categorical variables are imputed using the mode. For sensitivity analysis, missing symptom severity data in lost‐to‐follow‐up patients were first handled using multiple imputation (MI) before performing LCGM. This was followed by a secondary LCGM analysis, restricted to patients who completed all three follow‐up visits, to verify model stability.

#### 2.5.2 Symptom Cluster Extraction and Comparison

A low prevalence of symptoms may interfere with the analytical process; therefore, symptoms with a prevalence ≥ 10% were included in the statistical inference [37]. Principal component analysis, combined with the maximum variance orthogonal rotation method, was used to analyze the symptom cluster. The Kaiser–Meyer–Olkin (KMO) test (KMO > 0.6) and Bartlett's test of sphericity (*p* < 0.05) were used to assess the suitability of the data for further EFA. Cronbach's *α* coefficient was used to determine the internal consistency of symptom clusters. Furthermore, descriptive comparisons were used to show the distribution and composition of the symptom clusters at different times, and a nonparametric test was used to compare the severity within the same symptom group before and after.

#### 2.5.3 Trajectory Analyses

LCGM in Mplus was conducted to identify trajectories for the MDASI‐GI overall scores in patients with CRC during the 3 months postoperatively. Given the assumption of individual heterogeneity within a population, LCGM characterizes this heterogeneity by partitioning the population into several mutually exclusive latent subpopulations. This model can fit the developmental trajectory lines of multiple categories, and individuals within a single trajectory are considered homogeneous. The difference in the intercept reflects the difference in the baseline symptom cluster, whereas the difference in the slope reflects the difference in the rate of symptom cluster development over time [38]. Model fit was assessed using multiple indices: the likelihood ratio chi‐square test (*G* ^2^ (LL)), Akaike Information Criterion (AIC), Bayesian Information Criterion (BIC), and sample size‐adjusted BIC (aBIC) [39], where lower values indicate better fit. Classification accuracy was evaluated using entropy (range 0–1), with values approaching 1 indicating superior classification accuracy (entropy = 0.8 corresponds to > 90% classification accuracy) [40]. Model selection was determined by bootstrapped likelihood ratio tests (BLRT) and Lo–Mendell–Rubin adjusted likelihood ratio tests (LMR) (*p* < 0.05, indicating statistically significant improvement for n‐class versus [*n*−1]‐class solutions). When indices conflicted, the optimal number of classes was determined by considering clinical relevance and ensuring that each subgroup comprised ≥ 5% of the sample, consistent with established methodological standards [39].

#### 2.5.4 Influencing Factor Analyses

Influencing factor analysis was performed in SPSS. The *t*‐test and chi‐square test were used to compare the demographic and clinical characteristics of patients with CRC under different symptom cluster trajectories. Binomial logistic regression analysis was used to identify the predictors of different trajectory categories. Two‐sided analyses with *p* < 0.05 were considered significant.

### 2.6 Ethical Approval

All procedures were performed in accordance with the Declaration of Helsinki of 1964 and subsequent versions. This study was approved by the Sichuan Provincial People's Hospital, School of Medicine, University of Electronic Science and Technology of China (No. 402, 2022). Participants received oral and written information before participating in the study and provided written informed consent.

## 3 Results

### 3.1 Sample Characteristics

A total of 203 patients were included in the study. The demographic and clinical characteristics of the patients are shown in Table 1. Due to rigorous quality control procedures implemented throughout the study, missing data were minimal for most variables, except for those from patients lost to follow‐up (Table S1).

**TABLE 1:** The demographic and clinical characteristics of patients (*N* = 203).

Variant	Class	Low symptoms—continuous decline	High symptom—decreases and then increases	*t*/ $\chi^{2}$	*p*
Sex	Male	102 (60.7%)	19 (54.3%)	0.497	0.481
	Female	66 (39.3%)	16 (45.7%)		
Age (years)	< 60	66 (39.3%)	13 (37.1%)	0.056	0.813
	≥ 60	102 (60.7%)	22 (62.9%)		
BMI	Normal	86 (51.2%)	20 (57.1%)	0.411	0.521
	Abnormal	82 (48.8%)	15 (42.9%)		
**Smoking**	No	105 (62.5%)	28 (80%)	**3.926**	**0.048**
	Yes	63 (37.5%)	7 (20%)		
Alcoholism	No	125 (74.4%)	31 (88.6%)	3.267	0.071
	Yes	43 (25.6%)	4 (11.4%)		
Educational level	Primary school or under	56 (33.3%)	17 (48.6%)	3.137	0.371
	Junior high school education	56 (33.3%)	9 (25.7%)		
	High school education	30 (17.9%)	4 (11.4%)		
	Bachelor's degree or above	26 (15.5%)	5 (14.3%)		
Spouse	No	24 (14.3%)	5 (14.3%)	0.000	1.000
	Yes	144 (85.7%)	30 (85.7%)		
Number of children	0	5 (3.0%)	1 (2.9%)	1.029	0.834
	1–2	136 (81.0%)	31 (88.6%)		
	3–4	24 (14.3%)	3 (8.6%)		
	≥ 5	3 (1.8%)	0 (0.0%)		
Living environment	Living alone	17 (10.1%)	3 (8.6%)	0.232	0.891
	Living with spouse	123 (73.2%)	27 (77.1%)		
	Living with others	28 (16.7%)	5 (14.3%)		
Regular place of residence	City	113 (67.3%)	26 (74.3%)	0.662	0.416
	Village	55 (32.7%)	9 (25.7%)		
Medical insurance	No	1 (0.6%)	0 (0.0%)	1.000	0.828
	Yes	167 (99.4%)	35 (100.0%)		
Career	Mainly physical labor	98 (58.3%)	21 (60.0%)	0.033	0.855
	Mainly mental labor	70 (41.7%)	14 (40.0%)		
Monthly per capita household income (CNY)	≤ 3000	68 (40.5%)	14 (40.0%)	0.336	0.953
	3000–4999	59 (35.1%)	11 (31.4%)		
	5000–9999	32 (19.0%)	8 (22.9%)		
	≥ 10,000	9 (5.4%)	2 (5.7%)		
**Perceived health status**	Rare	30 (17.9%)	4 (11.4%)	**14.494**	**0.004**
	Good	55 (32.7%)	17 (48.6%)		
	General	64 (38.1%)	5 (14.3%)		
	Not so good	17 (10.1%)	6 (17.1%)		
	Very poor	2 (1.2%)	3 (8.6%)		
**Combination of multiple chronic diseases (No. ≥ 2)**	No	107 (63.7%)	14 (40.0%)	**6.752**	**0.009**
	Yes	61 (36.3%)	21 (60.0%)		
Combined high blood pressure	No	126 (75.0%)	22 (62.9%)	2.162	0.141
	Yes	42 (25.0%)	13 (37.1%)		
Combined diabetes	No	152 (90.5%)	33 (94.3%)	0.156	0.693
	Yes	16 (9.5%)	2 (5.7%)		
Combined heart disease	No	153 (91.1%)	29 (82.9%)	1.315	0.252
	Yes	15 (8.9%)	6 (17.1%)		
**Combined chronic lung disease**	No	162 (96.4%)	29 (82.9%)	**7.307**	**0.007**
	Yes	6 (3.6%)	6 (17.1%)		
Combined central nervous system disorders	No	161 (95.8%)	31 (88.6%)	1.732	0.188
	Yes	7 (4.2%)	4 (11.4%)		
Surgical history	No	102 (60.7%)	19 (54.3%)	0.497	0.481
	Yes	66 (39.3%)	16 (45.7%)		
Tumor position	Colon	74 (44.0%)	14 (40.0%)	0.435	0.800
	Rectum	92 (54.8%)	2 (60.0%)		
	Colorectal	2 (1.2%)	0 (0.0%)		
Cancer staging	Stage 1	15 (8.9%)	3 (8.6%)	1.412	0.842
	Stage 2	32 (19.0%)	5 (14.3%)		
	Stage 3	54 (32.1%)	14 (40.0%)		
	Stage 4	57 (33.9%)	12 (34.3%)		
	Indefinite	10 (6.0%)	1 (2.9%)		
Pathological diagnosis	Adenocarcinoma	152 (90.5%)	28 (80.0%)	6.618	0.066
	Mucous membrane cancer	8 (4.8%)	3 (8.6%)		
	Others	2 (1.2%)	3 (8.6%)		
	Indefinite	6 (3.6%)	1 (2.9%)		
Degree of differentiation	Low polarization	12 (7.1%)	0 (0.0%)	3.159	0.353
	Medium polarization	103 (61.3%)	26 (74.3%)		
	High polarization	29 (17.3%)	5 (14.3%)		
	Indefinite	24 (14.3%)	4 (11.4%)		
**Type of surgery**	Laparoscopic surgery	140 (83.3%)	23 (65.7%)	**5.683**	**0.017**
	Open surgery	28 (16.7%)	12 (34.3%)		
Radical surgery	No	19 (11.3%)	6 (17.1%)	0.452	0.501
	Yes	149 (88.7%)	29 (82.9%)		
Stoma	No	127 (75.6%)	21 (60.0%)	3.566	0.059
	Yes	41 (24.4%)	14 (40.0%)		
Anemic	No	75 (44.6%)	14 (40.0%)	0.254	0.615
	Yes	93 (55.4%)	21 (60.0%)		
Intraoperative blood transfusion	No	159 (94.6%)	31 (88.6%)	0.912	0.339
	Yes	9 (5.4%)	4 (11.4%)		
Albumin	≥ 35 g	13 (37.1%)	66 (39.3%)	0.056	0.813
	< 35 g	22 (62.9%)	102 (60.7%)		
Postoperative complication	No	139 (82.7%)	24 (68.6%)	3.674	0.055
	Yes	29 (17.3%)	11 (31.4%)		
Postoperative admission to ICU	No	157 (93.5%)	30 (85.7%)	1.442	0.230
	Yes	11 (6.5%)	5 (14.3%)		
**Postoperative chemotherapy**	No	145 (86.3%)	21 (60.0%)	**13.452**	**0.000**
	Yes	23 (13.7%)	14 (40.0%)		
**Intensity of preoperative physical activity**	Low	6 (3.6%)	5 (14.3%)	**7.447**	**0.024**
	Medium	121 (72.0%)	25 (71.4%)		
	High	41 (24.4%)	5 (14.3%)		
**Preoperative frailty**	No	147 (87.5%)	25 (71.4%)	**5.782**	**0.016**
	Yes	21 (12.5%)	10 (28.6%)		
**Severe preoperative anxiety and depression**	No	131 (78.0%)	21 (60.0%)	**4.976**	**0.026**
	Yes	37 (22.0%)	14 (40.0%)		
Nutritional risks (NRS‐2002)	No	26 (15.5%)	3 (8.6%)	1.128	0.288
	Yes	142 (84.5%)	32 (91.4%)		
ASA classification	1~2分	133 (79.2%)	26 (74.3%)	0.406	0.524
	3~4分	35 (20.8%)	9 (25.7%)		
Surgical risk assessment	0分	24 (14.3%)	3 (8.6%)	0.829	0.661
	1分	118 (70.2%)	26 (74.3%)		
	2分	26 (15.5%)	6 (17.1%)		

*Note:* Bold fonts indicate *p* < 0.05.

### 3.2 Composition and Changes in Symptom Clusters at Different Times

The severity of symptoms was not normally distributed. Six symptom clusters were extracted by EFA at time points T1–T3 (Table 2). Comparing the changes in the severity of symptom clusters at different time points, the average score of symptom cluster severity was the overall sum of symptom severity in each symptom cluster divided by the number of symptom items in the group (Table 3).

**TABLE 2:** Composition and comparison of symptom clusters at different postoperative time points in patients with colorectal cancer.

1‐Level symptom cluster	2‐Level symptom cluster	T1 (*n* = 203)		T2 (*n* = 164)		T3 (*n* = 139)	
		Symptom	Factor loading	Symptom	Factor loading	Symptom	Factor loading
Spiritless and psychological symptom cluster	Mood‐sleep disorder symptom cluster	Distressed	0.834	—	—	—	—
		Sadness	0.821	—	—	—	—
		Disturbed sleep^a^, ^b^	0.570	—	—	—	—
		Abdominal^b^ distention	0.519	—	—	—	—
		Variance explained	32.0%				
		Cronbach's *α*	0.721				
	Nervous system and spiritless symptom cluster	Sleepy	0.729	—	—	—	—
		Memory decline	0.700	—	—	—	—
		Shortness of breath^a^	0.522	—	—	—	—
		Variance explained	9.9%				
		Cronbach's *α*	0.517				
Pain symptom cluster	Pain‐physical symptom cluster	Nausea	0.721	—	—	—	—
		**Pain** ^a^, ^b^	0.652	—	—	—	—
		Appetite loss	0.571	—	—	—	—
		Dry mouth^a^, ^b^	0.538	—	—	—	—
		Fatigue^a^, ^b^	0.457	—	—	—	—
		Variance explained	11.1%				
		Cronbach's *α*	0.647				
	Pain‐psychological symptom cluster	—	—	**Pain** ^a^, ^b^	0.764	—	—
		—	—	Abdominal distention^a^	0.663	—	—
		—	—	Disturbed sleep^a^, ^b^	0.615	—	—
		—	—	Distressed	0.575	—	—
				Variance explained	12.5%		
				Cronbach's *α*	0.638		
Sickness behavior symptom cluster	Activity intolerance symptom cluster	—	—	**Fatigue** ^a^, ^b^	0.827	**Shortness of breath** ^a^	0.923
		—	—	**Shortness of breath** ^b^	0.770	**Fatigue** ^a^, ^b^	0.794
		—	—	Dry mouth	0.652	—	—
		—	—	**Appetite loss** ^a^, ^b^	0.648	—	—
				Variance explained	42.7%	Variance explained	53.3%
				Cronbach's *α*	0.759	Cronbach's *α*	0.749
	Lack of energy symptom cluster	—	—	—	—	Disturbed sleep^a^, ^b^	0.871
		—	—	—	—	**Appetite loss** ^a^, ^b^	0.687
						Variance explained	21.9%
						Cronbach's *α*	0.455

*Note:* (1) At T1, 13 symptoms had an incidence of ≥ 10%. A total of four factors were extracted in the first round of exploratory factor analysis. Factor 4 included diarrhea and abdominal distension symptoms but had a low Cronbach's *α* (0.108), indicating poor internal consistency. Since diarrhea was only associated with Factor 4, it was excluded, and the analysis was repeated. The revised results identified three distinct factors. (2) Eight symptoms at T2 had a prevalence of ≥ 10%. (3) At T3, six symptoms had an incidence of ≥ 10%, and diarrhea and pain were excluded from the symptom cluster EFA analysis. (4) Black bold font indicates the same symptoms within the symptom group.

^a^ The prevalence of this symptom is in the top five.

^b^ The severity of the symptom is ranked in the top five.

**TABLE 3:** Longitudinal changes in the severity of overall symptom clusters in patients with colorectal cancer 3 months after surgery.

Symptom cluster	T1	T2	T3	|△Symptom cluster severity|	*Z*/*c* ^2^	*p*
	($\bar{x}\pm s$, score)	($\bar{x}\pm s$, score)	($\bar{x}\pm s$, score)	($\bar{x}\pm s$, score)		
Activity intolerance symptom cluster		0.51 ± 0.96	0.39 ± 0.83	0.16 ± 0.98	−1.57	0.12
Pain symptom cluster	2.11 ± 1.30	0.57 ± 0.85		1.55 ± 1.54	−9.61	0.00*
Sickness behavior symptom cluster		0.51 ± 0.96	0.48 ± 0.79	0.03 ± 0.99	−0.02	0.99
Overall symptom clusters	1.50 ± 1.01	0.55 ± 0.82	0.48 ± 0.79	0.95 ± 1.29	126.99	0.00*
				0.06 ± 0.87		

*Note:* (1) | | Absolute value symbol. (2) △ Difference symbol. (3) Bold fonts represent significant *p*‐values. (4) Overall symptom cluster. (5) The severity of the overall symptom cluster was compared for post hoc analysis, and *p*‐values were adjusted using the Bonferroni correction method. The test results indicated significant differences in the overall symptom cluster scores between T1 and T2 or between T1 and T3 (all p* < 0.05), whereas there were no significant differences in the overall symptom cluster severity between T2 and T3 (p* > 0.05).

### 3.3 Trajectory Analysis of the Overall Symptom Clusters

**FIGURE 2:**
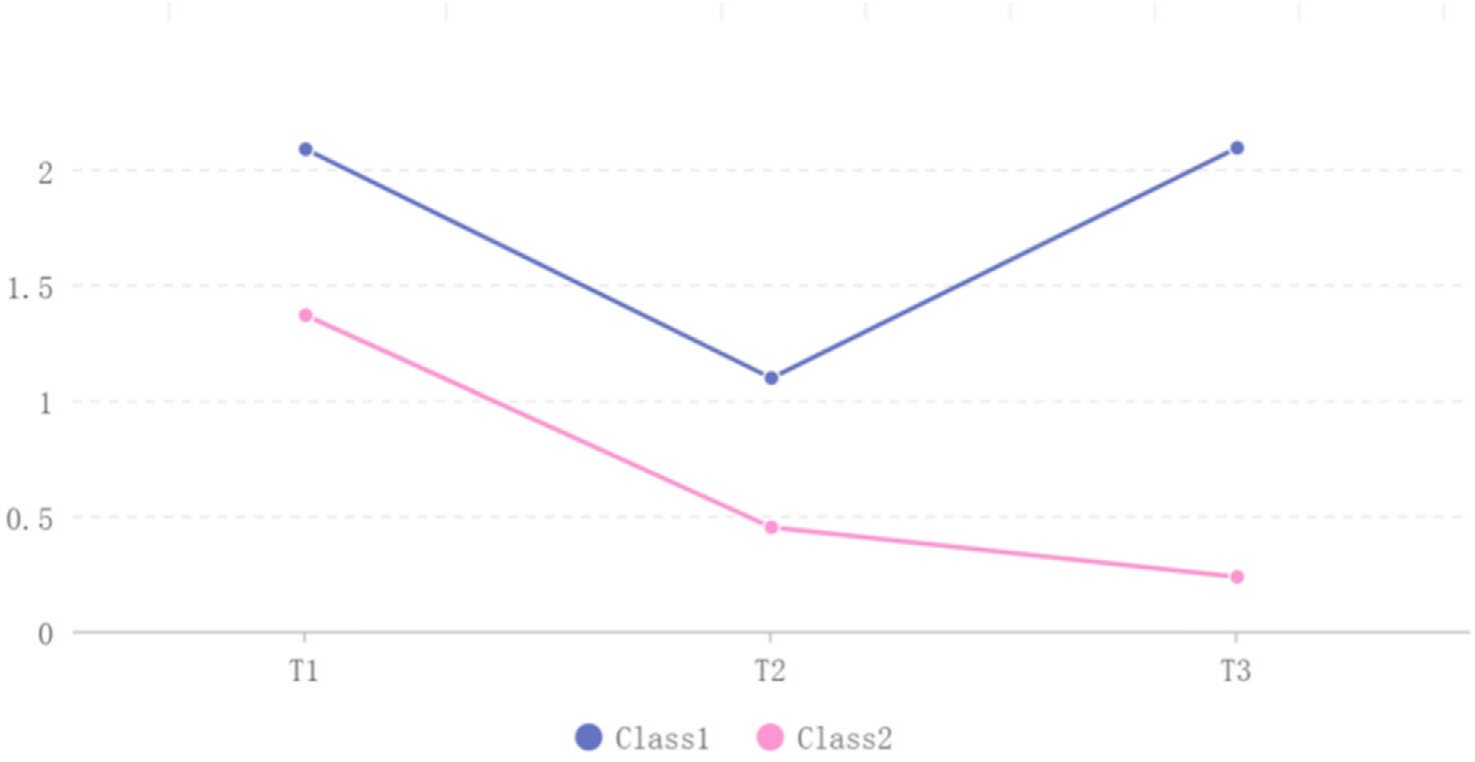
Heterogeneous trajectories of the mean severity of overall symptom clusters in patients with CRC 3 months postoperative. Class 1 represents a trend of “high symptom—decreases and then increases,” and Class 2 represents a trend of “low symptoms—continuous decline.”

Since clusters of 1‐level and 2‐level symptoms were observed at no more than two time points, the heterogeneity in the overall symptom cluster was investigated. Using the LCGM and clinical interpretability identified model 2 (BIC less than one category. Entropy=0.916. Both LMR and BLRT were statistically significant, and the class probabilities were both greater than 5%) was identified as the best‐fitting model (Table 4). The population was divided into two categories based on the initial symptom level and trajectory characteristics: “high symptom—decreases and then increases” (17.2%) and “low declining type” (82.8%) (Figure 2).

**TABLE 4:** Summary of information on the fitting of latent class growth models for overall symptom clusters in patients with colorectal cancer (*N* = 203).

Model	*K*	*G* ^2^ (LL)	AIC	BIC	aBIC	Entropy	LMR	BLRT	Class probability
1C	5	−788.481	1586.962	1603.528	1587.687	—	—	—	1
**2C**	**8**	**−742.251**	**1500.503**	**1527.008**	**1501.662**	**0.916**	**0.028**	**0.000**	**0.172/0.828**
3C	11	−715.503	1453.006	1489.451	1454.601	0.947	0.001	0.000	0.788/0.168/0.044
4C	14	−703.460	1434.921	1481.305	1436.950	0.915	0.226	0.000	0.108/0.197/0.039/0.655

*Note:* The bold values represent the optimal model.

Sensitivity analysis revealed that the results obtained using the deletion method (Table S2) were consistent with those derived from the MI method (Table 4). This suggests that the impact of loss to follow‐up, attributable to various reasons, on the heterogeneous trajectory of the overall symptom clusters is negligible.

### 3.4 Analysis of Influencing Factors of Overall Symptom Clusters Trajectory

**FIGURE 3:**
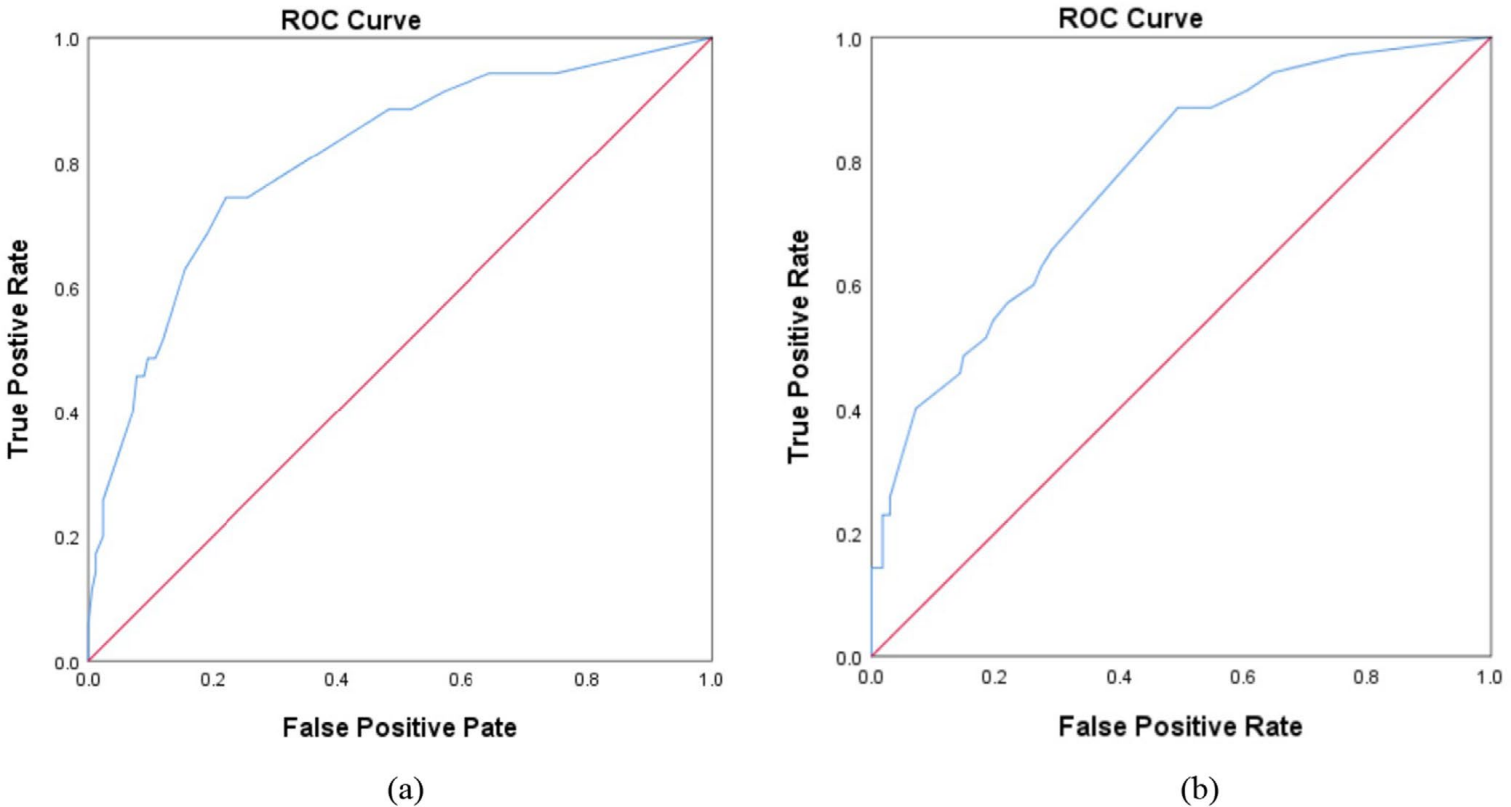
(a) Area under the ROC curve of Model I with the AUC is 0.808. (b) Area under the ROC curve of Model II with the AUC of 0.772.

Further evaluate the nine statistically significant variables identified in the univariate analysis (Table 1). Univariate binary logistic regression analysis excluded smoking and perceived health status (*p* < 0.05). In order to improve clinical applicability, we established two prediction models according to different nursing scenarios. Both models adopted the forward stepwise selection method (likelihood ratio) for variable inclusion. In Model I, the initial selection only considers variables observed during the pre‐rehabilitation or perioperative period. The final predictors include severe preoperative anxiety and depression, a combination of multiple chronic diseases (No. ≥ 2), combined chronic lung disease, and the type of surgery. Model II expands the initial variable pool by incorporating postoperative chemotherapy (a non‐universal treatment) to consider the management during the continuous treatment phase. The final predictors of Model II include preoperative frailty, severe preoperative anxiety and depression, combined chronic lung disease, and postoperative chemotherapy. The prediction accuracy rates of Models 1 and were 84.2% and 85.2%, respectively. Six key “high symptom—decreases and then increases” risk predictors were successfully identified (Table 5, Figure 3).

**TABLE 5:** Factors influencing the trajectory of overall symptom clusters in participants (*N* = 203).

Variant	Univariate analysis		Model I: multivariate analysis		Model II: multivariate analysis		Collinearity diagnostics	
	OR (95% CI)	*p*	OR (95% CI)	*p*	OR (95% CI)	*p*	Tolerance	Variance inflation factor
Smoking^a^	0.42 (0.17–1.01)	0.053						
Perceived health status^a^	1.21 (0.84–1.76)	0.303						
Intensity of preoperative physical activity^b^, ^c^	0.41 (0.19–0.91)	**0.028**					0.859	1.164
Preoperative frailty^b^, ^c^	2.8 (1.18–6.65)	**0.020**			3.12 (1.21–8.05)	**0.019**	0.846	1.183
Severe preoperative anxiety and depression^b^, ^c^	2.36 (1.10–5.09)	**0.028**	2.87 (1.26–6.56)	**0.040**	2.43 (1.05–5.64)	**0.039**	0.979	1.021
Combination of multiple chronic diseases (No. ≥ 2)^b^, ^c^	2.63 (1.25–5.55)	**0.011**	2.41 (1.04–5.55)	**0.037**			0.873	1.145
Combined chronic lung disease^b^, ^c^	5.59 (1.69–18.52)	**0.005**	4.07 (1.09–15.25)	**0.008**	6.80 (1.85–25.03)	**0.004**	0.903	1.108
Type of surgery^b^, ^c^	2.61 (1.16 ~ 5.85)	**0.020**	3.26 (1.37–7.74)	**0.012**			0.925	1.081
Postoperative chemotherapy^c^	4.20 (1.88–9.42)	**0.000**			5.47 (2.26–13.26)	**0.000**	0.945	1.058

*Note:* Bold fonts indicate *p* < 0.05.

^a^ Variable excluded by univariate binary logistic regression.

^b^ Variable initially selected by Model 1.

^c^ Variable initially selected in Model 2.

## 4 Discussion

### 4.1 Focusing on Changes in Symptom Clusters During Postoperative Recovery to Provide a Basis for Saving Healthcare Resources

Patients with CRC experience a variety of symptoms during the initial 3 months post‐surgery. While the incidence and severity of most symptoms gradually decrease over time, pain, fatigue, sleep disturbance, dyspnea, and loss of appetite remain prevalent throughout T1, T2, and T3, with an incidence of > 10%. Several studies also identified these symptoms at different times in patients with CRC [2, 19, 41], and these symptoms were identified in different combinations in a larger cluster of cancer‐related symptoms [42]. Therefore, these five symptoms are the most prevalent, stable, and clinically significant symptoms in patients with CRC after surgery and require extra attention from medical staff.

The reliability analysis revealed acceptable internal consistency (Cronbach's *α* = 0.517–0.759) for five symptom clusters in this exploratory factor analysis, while the energy deficiency cluster (disturbed sleep and appetite loss) showed marginally lower consistency (*α* = 0.455). This observation is consistent with prior research documenting comparable suboptimal reliability coefficients, including a Cronbach's *α* of 0.497 for a sensory disturbance symptom cluster among post‐radiotherapy nasopharyngeal carcinoma survivors (*n* = 130) [43] and 0.43 for a chemotherapy‐induced neurotoxicity cluster in gastrointestinal cancer patients (*n* = 212) [44]. These marginally acceptable values may reflect methodological limitations such as restricted sample sizes or reduced symptom heterogeneity within the identified clusters. Future exploratory studies should incorporate larger, multicenter cohorts to enhance the stability of symptom cluster identification and validation.

In this study, the distribution of symptom clusters varied, yet remained relatively stable, consistent with other findings. Notably, patients were more affected by the mood‐sleep disorder symptom cluster 7 days after surgery and by the activity intolerance symptom cluster 1.5–3 months postoperatively. Targeted interventions for these clusters may yield greater benefits for patients. In this study, patients from southwestern China exhibited a more homogeneous distribution of symptom clusters within the first 3 months compared to patients from other regions of China. Although foreign CRC patients exhibit similar symptoms during this period, differences exist in symptom cluster distribution and their impact on quality of life. Additionally, the subjective symptoms reported by patients may vary due to cultural influences [14]. The participants in this study were from southwest China, and their distribution of cross‐sectional symptom clusters within 3 months was more similar compared to other regions in China [2]. While we identified similar symptoms among patients with CRC internationally during this period [19, 20], the distribution of symptom clusters and their potential impact on quality of life exhibited slight variations. Consequently, national or regional differences should be taken into account when designing targeted interventions.

### 4.2 “High Symptom—Decreases and Then Increases” Group: Higher Overall Symptom Cluster Levels at All Time Points, Requiring More Focused Attention

The mixture modeling techniques demonstrate flexibility, accuracy, and high resolution for identifying population trends and differentiating clinical subgroups [38]. The LCGM analysis identified two distinct trajectories of postoperative symptom clusters in patients with CRC (Figure 2): “high symptom—decreases and then increases” (17.1%) and “low symptoms—continuous decline” (82.9%). The latter group demonstrated stable symptom resolution, with faster improvement during the first 6 weeks, followed by gradual decline to 3 months, consistent with typical postoperative recovery patterns in patients with CRC [2, 19]. In contrast, the “high symptom—decreases and then increases” group maintained higher symptom severity than the “low symptoms—continuous decline” group at all timepoints. This divergence may reflect differences in surgical factors, baseline health status, and psychological distress [45, 46, 47]. The observed symptom rebound after 6 weeks likely corresponds to the initiation of adjuvant chemotherapy. Although comprising only 17.1% of patients, this high‐risk subgroup needs to strengthen the management of the “spiritless and psychological symptom clusters” and the “pain symptom clusters” during the perioperative period, and implement precise rehabilitation care or home care for the “sickness behavior symptom cluster” after discharge. Early identification of risk factors can enable targeted intervention measures to optimize resource allocation and improve outcomes.

Notably, both symptom clusters exhibited persistent sickness behavior at the 3‐month follow‐up. The underlying pathophysiology may involve tumor microenvironment‐induced chronic inflammation mediated through the cytokine‐neural axis (proinflammatory cytokines → CNS → peripheral nerves) [48]. An anti‐inflammatory diet and appropriate exercise can reduce chronic inflammation and improve health [49, 50]. Further research is needed to investigate the impact of diet and physical activity on inflammation in patients with CRC. Postoperative symptom monitoring in these patients should be extended to collect clinically relevant data.

### 4.3 Six Variables Predict the Trajectory of a “High Symptom—Decreases and Then Increases,” Suggesting Different Management Strategies for Different Periods

Patients with CRC exhibiting severe anxiety and depression or frailty before surgery were 2.43 and 3.12 times more likely to experience a “high symptom—decreases and then increases” trajectory 3 months postoperatively, compared to those without such conditions, respectively. Anxiety and depression impair treatment adherence and correlate strongly with poor prognosis, higher adverse event rates, and reduced survival in cancer patients [51, 52]. A meta‐analysis showed that approximately 30%–40% of patients with cancer in hospitals suffer from anxiety or depressive mood disorders, a significantly higher prevalence than in the general population [53]. To date, researchers have studied the relationship between anxiety‐depressive states and symptom clusters. Findings indicate that anxiety and depression are critical factors influencing symptom clusters [46]. Conversely, the exacerbation of symptom clusters can lead to an increase in the degree of anxiety–depression [54]. In addition, anxiety–depression plays a mediating role in the effect of symptom clusters on other variables [55]. Some studies have included anxiety and depression as components of symptom clusters [13]. Ye et al. [56] emphasized that emotional symptom clusters are core clusters during cancer treatment intervals, and managing them can significantly alleviate other symptoms. This shows the importance of anxiety and depression in symptom management. Preoperative frailty increases complication rates in patients, is independently associated with 30‐day mortality and long‐term survival [57, 58, 59]. In previous studies, the prevalence of frailty in patients with CRC ranges from 2% to 32%, while the prevalence of pre‐frailty ranges from 52% to 67% [60], which is consistent with the results in this study. As the global population ages, the disease burden among frail CRC populations is escalating. Prehabilitation, a preoperative management strategy based on ERAS, has successfully reduced postoperative complications, improved postoperative function, and reduced overall healthcare costs [61, 62]. Psychological interventions and frailty management are often important aspects of prehabilitation [61]. Currently, the management of frailty involves a comprehensive intervention that encompasses nutrition, exercise, and psychology [63, 64]. The psychological intervention component includes elements such as psychoeducation, cognitive‐behavioral therapy, and supportive psychotherapy. The primary aim is to help patients develop coping skills for managing negative emotions, enhance their self‐efficacy, and foster their enthusiasm for treatment [65, 66, 67]. Prior studies have indicated that preoperative pre‐rehabilitation based on this comprehensive approach can improve outcomes in debilitated populations. This study proposes that pre‐rehabilitation for patients with CRC experiencing severe anxiety, depression, or frailty before surgery may result in a positive trajectory of their overall symptom cluster 3 months post‐surgery. It is recommended to identify and address anxiety, depression, or frailty as early as possible following diagnosis and to incorporate these interventions into medium‐ and long‐term postoperative rehabilitation plans.

In this study, comorbid chronic lung disease and comorbid multiple chronic diseases (≥ 2) were influential factors in the “high symptom—decreases and then increases” subgroup. Analysis showed that patients with chronic lung disease had a 4–6 times higher risk of developing a “high symptom—decreases and then increases” trajectory compared to those without it. Chronic lung disease is an important factor affecting cardiopulmonary reserve, resulting in decreased cardiopulmonary function and activity tolerance, and is also an independent risk factor for postoperative complications and death [68]. Pulmonary rehabilitation training has been effective in improving lung function for patients with chronic lung disease and enhancing postoperative outcomes for patients undergoing pulmonary surgery; however, only a few studies have applied it to patients with CRC who also have chronic lung disease [69]. While pulmonary rehabilitation is primarily a long‐term health behavior that improves the physical condition of patients with chronic lung disease, the methods to enhance cardiopulmonary reserve function in patients during the prehabilitation stage or a short period during the perioperative period require further research. Patients with CRC with two or more comorbid chronic diseases were 2.41 times more likely to have a “high symptom—decreases and then increases” trajectory postoperatively than those without combined chronic diseases. Notably, in addition to comorbid chronic lung disease, patients with hypertension, diabetes, cardiac disease, and central nervous system disease alone did not show a significant difference in the overall symptom clusters postoperatively compared to patients with no comorbid disease. Multimorbidity is a growing global issue associated with aging. As the number of comorbidities increases, so does the cost of care and the risk of death [47, 70]. This study proposes that individuals should be considered as holistic entities, emphasizing their overall subjective experience rather than isolated indicators. Furthermore, clinical focus should be placed on patients with CRC with comorbidities, and active exploration of short‐term strategies to enhance cardiopulmonary function is warranted, which may alleviate the overall self‐reported symptoms experienced by patients post‐surgery.

We found that patients undergoing open surgery and postoperative chemotherapy were 3.26 and 5.47 times more likely to be classified as a “high symptom—decreases and then increases” subgroup than patients undergoing laparoscopic surgery and non‐chemotherapy, respectively. The inflammatory immune response is a classic explanation for the body's stress response: greater trauma leads to stronger stress responses [71]. Compared with open surgery, minimally invasive techniques can reduce incisional damage and decrease the body's inflammatory response, which in turn reduces postoperative complications, shortens the hospitalization period, and reduces the incidence of rehospitalization [45]. With the advancement of technology, minimally invasive surgery is expected to become the predominant approach, significantly enhancing patients' postoperative quality of life. Surgery combined with radiotherapy can improve the therapeutic effect in patients with CRC; however, the double stimulation of surgery and radiotherapy amplifies the risk of imbalance of the body's neuroendocrine and immune systems [72], which further aggravates the symptom burden of patients. It is advisable to promptly identify patients eligible for postoperative combination chemotherapy, investigate the distribution and trends of symptom clusters during chemotherapy, and establish a comprehensive whole‐course management plan to mitigate negative experiences. In future research, treatment and management strategies should be designed based on the underlying mechanisms of symptom clustering.

### 4.4 Strengths and Limitations

This study has several limitations that should be considered. The single‐center design, while controlling for inter‐institutional heterogeneity, may affect the generalizability of findings. Convenience sampling could introduce selection bias, and reliance on self‐reported data may be subject to psychological influences. Although we included core nutritional indicators (NRS‐2002, serum albumin, BMI), the omission of additional parameters (prealbumin, skinfold thickness, etc.) and the use of demographic‐based rather than scale‐based social support assessment may impact the accuracy of the results. The 3‐month follow‐up period, although providing preliminary longitudinal data, may be insufficient to fully capture the evolution of symptom clusters. Future research should incorporate more comprehensive assessments, extend follow‐up duration, and employ multicenter designs with rigorous sampling methods. Notably, the innovation point of this study lies in: successfully expanding the application of the LCGM in the dynamic evolution of non‐preset symptom clusters, providing a new methodological perspective for symptom management research. These findings warrant verification through larger‐scale, clinically stratified studies to develop more targeted intervention strategies.

## 5 Conclusion

The clinical significance of this study lies in its potential to guide early, targeted interventions for CRC patients, particularly those in the “high symptom—decreases and then increases” subgroup. Patients with chronic comorbidities, preoperative frailty, preoperative severe anxiety and depression, or those undergoing open surgery and chemotherapy may benefit from intensified management during the crucial 3‐month postoperative period, helping to alleviate symptom burden and improve recovery. By identifying symptom trajectories early, healthcare providers can tailor postoperative care strategies, prioritize follow‐up for high‐risk patients, and optimize resource allocation. This study emphasizes the importance of integrating symptom cluster management into long‐term care for CRC patients, which could improve patient outcomes and reduce healthcare costs. Addressing key symptom clusters like mood‐sleep disorder and activity intolerance through prehabilitation and specialized care may significantly enhance quality of life and foster better physical and emotional recovery post‐surgery.

## Author Contributions

**Yue Li:** conceptualization, methodology, formal analysis, writing – original draft, data curation, investigation. **Wenwen Gan:** investigation, data curation, project administration, formal analysis. **Qin Mao:** writing – review and editing, formal analysis, data curation. **Hongying Wu:** resources, visualization, project administration, investigation. **Ting Cao:** investigation, data curation, formal analysis. **Haiyan Wu:** resources, supervision, writing – review and editing, funding acquisition. **Xiaorong Mao:** visualization, methodology, writing – review and editing.

## Conflicts of Interest

The authors declare no conflicts of interest.

## Supporting information


Data S1.

Data S2.

## Data Availability

Due to the nature of this research, participants of this study did not agree for their data to be shared publicly, so supporting data is not available.
