# Supplementary material for: Trajectories of Symptom Clusters and Their Predictive Factors in Patients With Colorectal Cancer 3 Months After Surgery: A Longitudinal Study
Source: Cancer Med. 2025 Jul 14;14(13):e71025. doi: 10.1002/cam4.71025 (PMC12257499; doi:10.1002/cam4.71025)
Supplement: Supplementary file 1 — Data S1. [file CAM4-14-e71025-s002.docx]

# 10. Supplementary Materials

**Supplementary Table 1** The proportion of missing data for all variables

| Variable | Missing ratio (%) | Variable | Missing ratio (%) | Variable | Missing ratio (%) |
| --- | --- | --- | --- | --- | --- |
| Sex | - | Postoperative admission to ICU | - | T2: Difficulty remembering | 18.70 |
| Age (y) | - | Postoperative chemotherapy | - | T2: Lack of appetite | 18.70 |
| BMI | - | Intensity of preoperative physical activity | - | T2: Drowsiness | 18.70 |
| Smoking | - | Preoperative frailty | - | T2: Dry mouth | 18.70 |
| Alcoholism | - | Severe preoperative anxiety and depression | - | T2: Sadness | 18.70 |
| Educational level | - | Nutritional risks（NRS-2002) | - | T2: Vomiting | 18.70 |
| Spouse | - | ASA classification | - | T2: Numbness | 18.70 |
| Number of children | - | Surgical risk assessment | - | T2: Constipation | 18.70 |
| Living environment | - | T1: Pain | - | T2: Diarrhea or watery stools | 18.70 |
| Regular place of residence | - | T1: Fatigue | - | T2: Difficulty swallowing | 18.70 |
| Medical insurance | - | T1: Nausea | - | T2: Change in taste | 18.70 |
| Career | - | T1: Disturbed sleep | - | T2: Bloated | 18.70 |
| Monthly per capita household income (CNY) | 1.48 | T1: Distress | - | T3: Pain | 16.30 |
| Perceived health status | - | T1: Shortness of breath | - | T3: Fatigue | 16.30 |
| Combination of multiple chronic diseases**（No.≥2）** | - | T1: Difficulty remembering | - | T3: Nausea | 16.30 |
| Combined high blood pressure | - | T1: Lack of appetite | - | T3: Disturbed sleep | 16.30 |
| Combined diabetes | - | T1: Drowsiness | - | T3: Distress | 16.30 |
| Combined heart disease | - | T1: Dry mouth | - | T3: Shortness of breath | 16.30 |
| Combined chronic lung disease | - | T1: Sadness | - | T3: Difficulty remembering | 16.30 |
| Combined central nervous system disorders | - | T1: Vomiting | - | T3: Lack of appetite | 16.30 |
| Surgical history | - | T1: Numbness | - | T3: Drowsiness | 16.30 |
| Tumor position | - | T1: Constipation | - | T3: Dry mouth | 16.30 |
| Cancer Staging | - | T1: Diarrhea or watery stools | - | T3: Sadness | 16.30 |
| Pathological diagnosis | - | T1: Difficulty swallowing | - | T3: Vomiting | 16.30 |
| Degree of differentiation | - | T1: Change in taste | - | T3: Numbness | 16.30 |
| Type of surgery | - | T1: Bloated | - | T3: Constipation | 16.30 |
| Radical surgery | - | T2: Pain | 18.70 | T3: Diarrhea or watery stools | 16.30 |
| Stoma | - | T2: Fatigue | 18.70 | T3: Difficulty swallowing | 16.30 |
| Anemic | - | T2: Nausea | 18.70 | T3: Change in taste | 16.30 |
| Intraoperative blood transfusion | - | T2: Disturbed sleep | 18.70 | T3: Bloated | 16.30 |
| Albumin | - | T2: Distress | 18.70 |  |  |
| Postoperative complication | - | T2: Shortness of breath | 18.70 |  |  |

**Supplementary Table 2** Summary of information on the fitting of latent class growth models for overall symptom clusters in patients with colorectal cancer (N=139)

| Model | K | G2(LL) | AIC | BIC | aBIC | Entropy | LMR | BLRT | Class probability |
| --- | --- | --- | --- | --- | --- | --- | --- | --- | --- |
| 1C | 5 | -547.889 | 1105.778 | 1120.450 | 1104.631 |  |  |  | 1 |
| **2C** | **10** | **-497.284** | **1010.568** | **1034.044** | **1008.734** | **0.952** | **0.0243** | **0.000** | **0.172/0.827** |
| 3C | 11 | -477.070 | 976.141 | 1008.420 | 973.618 | 0.966 | 0.0721 | 0.000 | 0.813/0.147/0.040 |
| 4C | 14 | -462.132 | 952.265 | 993.347 | 949.055 | 0.945 | 0.3500 | 0.000 | 0.10072/0.734/0.130/0.036 |
