# Supplementary material for: Trajectories of Symptom Clusters and Their Predictive Factors in Patients With Colorectal Cancer 3 Months After Surgery: A Longitudinal Study
Source: Cancer Med. 2025 Jul 14;14(13):e71025. doi: 10.1002/cam4.71025 (PMC12257499; doi:10.1002/cam4.71025)
Supplement: Supplementary file 2 — Data S2. [file CAM4-14-e71025-s001.docx]

*Reporting* *Recommendations for MLM/LGM*

| *Recommendation* | *Page No* |
| --- | --- |
| Theoretical formulation and data collection phase  *Guiding principle: The description of the research question, pertinent theoretical perspective and past literature should clearly be reflected in the formulation of the current study and data collection activities*. | |
| - Describe how the research question/theory of change is consistent with the explanatory and response measures. | *2-5* |
| - Communicate considerations of the theoretical perspective in terms of how it translates to the multilevel structure of the data. | *2-5* |
| - Speak to how consistent the ‘theory of change’ is with the number and spacing of repeated measures. | */* |
| - Indicate for the reader what confidence can be placed in the measures, namely whether their meaning could change and whether the measures are sensitive to the phenomena being monitored. | *6-7* |
| - Justify the sample size and report briefly on any power analyses conducted. | *5-6* |
| - Thoroughly describe all models to be tested, along with any supporting rationale. It may be most efficient to provide the equations for the models, or drawings of the models. Additionally, a table that describes the main features of each model or distinctions between models can be useful to the reader. | *2-5* |
| Data preparation phase  *Guiding principle: Readers should be fully informed of any data issues that could compromise the validity of inferences made from the analyses being reported.* | |
| - Provide useful summary statistics for measures, taking into account grouping and levels present within the data. Reported statistics can include means, standard deviations, skewness, kurtosis, minimum and maximum values, and correlations. | *9-20, Supplementary Material 1-9* |
| - Discuss what was done to satisfy the assumptions of the statistical technique, such as examination of data distributions and linearity among measures. | *9* |
| - Describe any efforts with regard to missing data, including a summary of the extent of missing data at the different levels and any findings regarding the mechanism(s) for missing data. | *8* |
| - Report any data transformations or imputations of missing data. | *8* |
| - Provide some statement or discussion indicating the degree to which any data considerations or possible violation of assumptions might affect the outcome of statistical tests. | */* |
| *Analysis decisions phase*  *Guiding principle: The reader should be well-informed of analysis decisions so he or she can make his or her own judgment about whether any analysis decisions might have affected inferences made in the paper.* | |
| - If the type of model utilized (LGM versus MLM) was chosen because it offered a particular analytic advantage, this should be clearly articulated. The choice of a multilevel method should in general be supported as well. | *26* |
| - Report the software name and version. | *8* |
| - Describe the coding of the time variable and the merits of the choice in terms of how it pertains to the theory of change and/or interpretation. | *7-8* |
| - Describe the trend(s) being tested. | *15-17* |
| - Indicate which estimation method was chosen, and when more than one is used for different aspects of the study, ensure this is clearly reported. | */* |
| - Describe the type of input matrix (for LGM) and any data considerations such as centering (for both LGM and MLM). | *14* |
| - Report the error structure being used in each analysis, that is, the degree to which errors are assumed to be independent or not independent. | */* |
| Model evaluation, respecification, and inference phase  *Guiding principle: Enough information should be reported regarding model evaluation, any re-specification, and interpretation such that the reader can form his or her own judgment about the relative merit of each model tested.* | |
| - The author should establish and communicate procedures and decision criteria for evaluating models being tested. | *15-17* |
| - Complete findings from any model comparison procedures, such as deviance tests, should be clearly reported. | *15-17* |
| - When discussing results of tests of trends, the model-implied trend should be compared to the actual (mean) trend for subjects. | / |
| - Any models that were tested, but not developed a priori, should be clearly labeled as such. | 22-23 |
| - Indicate whether reported parameters are standardized or unstandardized, and if standardized, report necessary information for the reader to be clear of how they were standardized. | / |
